# CEA-Ki-67- Pathologic Subtype: An Adjunct Factor for Refining Prognosis in Stage I Pulmonary Adenocarcinoma

**DOI:** 10.3389/fsurg.2022.853363

**Published:** 2022-04-25

**Authors:** Dongzhi Yu, Yanbin Sun, Michael A. McNutt, Shun Xu

**Affiliations:** ^1^Department of General Thoracic Surgery, The First Affiliated Hospital of China Medical University, Shenyang, China; ^2^Department of Pathology and Molecular Biology, School of Medicine and Research Institute, Peking University, Beijing, China

**Keywords:** carcinoembryonic antigen (CEA), Ki-67, pathologic subtype, lung adenocarcinoma (AC), prognosis

## Abstract

**Objectives:**

The prognosis for stage I pulmonary adenocarcinoma is generally good. However, some patients with stage I pulmonary adenocarcinoma have an unexpectedly poor outcome. This warrants consideration of adjunct markers. In this study, we analyze carcinoembryonic antigen, Ki-67, and a pathologic subtype in combination for prognostic evaluation of stage I pulmonary adenocarcinoma. These factors were selected for study as they have been shown to be individually associated with prognosis in many studies.

**Methods:**

A total of 650 patients with stage I pulmonary adenocarcinoma were investigated retrospectively. Each patient was re-staged using standard TNM criteria. Carcinoembryonic antigen (CEA) values were obtained from preoperative blood samples, and Ki-67 was evaluated with tumor tissue immunohistochemistry. Patient clinicopathologic characteristics, survival status, and date of death were obtained from medical records and telephone follow-up.

**Results:**

CEA > 4.4 ng/ml, Ki-67 > 13%, and a solid-micropapillary tumor growth pattern were each independent adverse prognostic markers for 5-year disease specific survival in stage I pulmonary adenocarcinoma. However, in combination, these 3 factors yielded a prognostic value (designated “CEA-Ki-67-pathologic subtype” value). Stage I pulmonary adenocarcinoma of low-risk CEA-Ki-67-pathologic subtype (CKP) value show biologic behavior similar to TNM stage IA1 tumors, while stage I tumors of high-risk CKP value are similar in prognosis to TNM stage II.

**Conclusion:**

The CKP value may be used as an adjunct to the TNM classification, which may yield a more accurately defined prognosis for cases of stage I pulmonary adenocarcinoma. CKP value may identify patients at higher risk who may benefit from adjuvant chemotherapy. Conversely, lower risk CKP values may support avoidance of chemotherapy.

## Introduction

The TNM classification is in common use for lung cancer staging to determine prognosis, assess tumor progression, and guide treatment through comprehensive evaluation of tumor size, presence or absence of lymph node metastasis, and distant metastasis (1). With the widespread use of high-resolution computed tomography (HRCT) and better contemporary understanding of lung cancer pathobiology, the rate of discovery of early-stage lung cancer is increasing. The TNM classification system, which was originally established in 1968, has also undergone concurrent refinement and improvement over the last 50 years, particularly in regard to T staging (2). However, patients with a given identical TNM stage do not infrequently show unexpectedly marked differences in prognosis. For example, some patients in the early stage of disease, according to the TNM classification, may experience recurrence or metastasis. In the 8th edition of the TNM classification, the average predicted 5-year overall survival rate of patients with stage I lung cancer is 78.6%, and TNM stage I is further subclassified as IA1: 92%; IA2: 83%; IA3: 77%; and IB:68%, which demonstrates differences in overall survival (3). We have identified cohorts of patients classified into the same stage grouping who were assigned an identical TNM subclassification, and yet showed marked differences in prognosis. This not only raises a question as to why prognosis differs among these individuals, but also calls for a search for means in addition to anatomical TNM staging that can be used to refine evaluation of tumor prognosis.

Differences in recurrence and prognosis which correspond to tumor subtype in stage I pulmonary adenocarcinoma have been demonstrated in previous studies (4). Tumor subtyping is carried out by microscopic evaluation. Lepidic subtype adenocarcinoma is associated with the most favorable prognosis of all the pulmonary adenocarcinoma subtypes, while the micropapillary (MIP) subtype shows the highest rate of recurrence. Even minor components of MIP are associated with recurrence (5, 6). This suggests that incorporation of histopathologic subtypes may increase accuracy of prognostic evaluation.

We selected Ki-67 and CEA for use in conjunction with adenocarcinoma subtype in our prognostic model. These markers have been used individually in clinical practice and have been shown to have prognostic value. Moreover, both are easy to evaluate clinically. Ki-67 is a biomarker that reflects tumor proliferative activity. This marker has been confirmed to have significant prognostic and predictive value in several types of cancer, such as breast cancer, prostate cancer, and colorectal cancer (7). Although its significance in pulmonary cancer has not been definitively established, many meta-analyses have shown that high Ki-67 values are associated with poor prognosis (8). CEA shows similar correspondence with prognosis. It is a glycoprotein that is involved in cell adhesion. Serum CEA reflects tumor load in patients and is a tumor marker for non-small-cell lung carcinoma (NSCLC) (9). A high CEA level portents poor prognosis in patients with stage I NSCLC undergoing surgery. It is also a risk factor for occult regional lymph node metastasis (10, 11).

However, previous studies which have evaluated the prognostic significance of CEA, Ki-67, and pathologic subtype in NSCLC have been qualitative analyses, without exactly defined cut-off points. Moreover, to the best of our knowledge there has been no study which has incorporated CEA, Ki-67, and pathologic subtype together for evaluation of prognosis in stage I lung adenocarcinoma.

This study is not intended to alter the TNM staging classification. Our focus instead is the evaluation of the potential adjunct prognostic significance of tumor CEA, Ki-67, and pathologic subtype, and to determine how to use these factors to refine accuracy of prognosis in patients who have surgery for TNM stage I pulmonary adenocarcinoma.

## Materials and Methods

### Ethical Approval of the Study Protocol

The Institutional Review Board of the First Affiliated Hospital of China Medical University approved this retrospective study (2021-99). Written informed consent from individual patients was waived because the study was a retrospective review.

### Study Design

We collected data on 2,654 patients with NSCLC who had undergone complete surgical resection in the Department of Thoracic Surgery of the First Affiliated Hospital of China Medical University from January 2014 to June 2016.

### Inclusion Criteria

Data on age, gender, tumor location, surgical procedure, patient survival status, and date of death were obtained through medical records and telephone follow-up. Tumor size, pathologic subtype, microvascular invasion, pleural invasion, lymph node status, and presence/absence of distant metastasis were obtained through the postoperative pathology report. CEA values were obtained by preoperative examination of blood, and Ki-67 values were obtained using postoperative immunohistochemistry on tumor tissue.

### Exclusion Criteria

Patients with incomplete clinicopathologic data were excluded from the study, and all pathologic data was confirmed by two pathologists. Other specific grounds for exclusion included preoperative neoadjuvant therapy, presence of microvascular invasion, a pathologic tumor type other than adenocarcinoma, or an adenocarcinoma *in situ* (AIS) pathologic subtype (adenocarcinoma *in situ*) or minimally invasive adenocarcinoma (MIA), or non-primary lung cancer. Patients who were lost to follow-up or died of other disease were not considered for further evaluation.

### Statistical Analysis

Ultimately, 650 patients with stage I pulmonary adenocarcinoma were analyzed retrospectively in this study. All patients underwent routine craniocerebral CT, pulmonary CT, abdominal CT, and bone scintigraphy (ECT) to evaluate for metastasis before surgery. If their CT reports show an existing problem, contrast enhancement CT scan, MRI, or biopsy was additionally done. They would then undergo VATS lobectomy and radical lymph node dissection.

The Kaplan–Meier method was used for analysis of the factors selected for evaluation. Log-rank tests were employed for determination of factors related to prognosis and estimation of their significance. Receiver operating characteristic (ROC) curves were used for analysis of CEA and Ki-67 as continuous variables and selection of potential cut-off points. Cyclooxygenase (Cox) proportional hazard models were used in multivariate analysis for the stratified assignment of factors putatively related to prognosis. Five-year disease specific survival (5-year DSS) was defined based on the group of patients who died from lung cancer within 5 years after surgery. Patients who died from causes other than lung cancer were not counted for this study. All tests were two-sided and performed using SPSS Statistics 25. A value of *p* < 0.05 was considered statistically significant.

## Histopathologic Evaluation

Resected specimens were formalin fixed, sectioned, and evaluated microscopically after staining with hematoxylin and eosin using standard techniques. Immunohistochemistry (IHC) was carried out with monoclonal Ki-67 antibody, and Ki-67 was evaluated with IHC and calculated as positive tumor cell staining in 5% increments.

All patients were re-staged according to the eighth edition of the TNM classification (12), and pathologic subtype was assigned according to the predominant histologic subtype as described in the 2015 WHO guidelines (13). Many previous studies have divided pathologic subtypes of pulmonary adenocarcinoma into lepidic predominant adenocarcinoma (LPA), papillary predominant adenocarcinoma or acinar predominant adenocarcinoma (PPA or APA), and solid or micropapillary predominant adenocarcinoma (SPA or MPA). In these studies, the pattern involving the highest percentage of tumor tissue was designated as the predominant pattern. Tumors were then categorized according to histopathologic findings as low-risk (LPA), intermediate-risk (PPA or APA), and high-risk (SPA or MPA).

In the course of clinical evaluation, we found that TNM stage I pathologic subtypes are ordinarily infrequently categorized as high-risk. In most tumors, even if there are solid (SOL) or micropapillary (MIP) components, these are typically only a minor component of the tumor and are not taken into account. Therefore, our current study presents a novel incorporation of evaluation of pulmonary adenocarcinoma based on pathologic subtype. Components of SOL and MIP, regardless of whether they are predominant components, are designated to be SOL-MIP positive. As a result, some tumors which would have been classified as LPA or PPA and APA in previous studies would be designated SOL-MIP positive according to our method of evaluation. According to previous research, we divided tumors into low-risk (LPA), intermediate-risk (PPA or APA), and high-risk (SOL-MIP positive) according to histopathologic findings.

## Results

### Clinicopathologic Features

Clinicopathologic characteristics of these 650 patients with TNM stage I lung cancer are summarized in Table 1. In brief, high-risk pathologic subtype, high CEA, and high Ki-67 values were more frequently found to be TNM stage IB rather than IA1, and these lesions were more frequently associated with patient mortality.

**Table 1:** Summary of patient cohort and characteristics in patients with stage I pulmonary adenocarcinoma (*n* = 650).

**Stage**	**IA1 (***n*** = 105)**	**IA2 (***n*** = 287)**	**IA3 (***n*** = 171)**	**IB (***n*** = 87)**
**Characteristics**				
**Gender (dead of disease)**				
Male	40 (3)	97 (17)	77 (19)	44 (14)
Female	65 (2)	190 (17)	94 (9)	43 (8)
**Age (dead of disease)**				
≤ 67 years	67 (2)	161 (15)	106 (15)	48 (12)
>67 years	38 (3)	126 (19)	67 (13)	39 (10)
**Tumor location (dead of disease)**				
RUL	30 (2)	95 (12)	65 (12)	36 (7)
RML	11 (1)	27 (6)	10 (0)	4 (2)
RLL	26 (1)	45 (0)	29 (7)	17 (7)
LUL	21 (1)	77 (12)	42 (5)	14 (2)
LLL	17 (0)	43 (4)	25 (4)	16 (4)
**Pathologic subtype (dead of disease)**				
Low-risk	41 (1)	73 (3)	29 (1)	6 (0)
Intermediate-risk	51 (2)	165 (17)	95 (12)	41 (8)
High-risk	13 (2)	49 (14)	47 (15)	40 (14)
**CEA stage (dead of disease)**				
X ≤ 4.4	97 (4)	242 (13)	126 (13)	42 (5)
4.4 < X ≤ 8.8	8 (1)	31 (12)	28 (7)	26 (6)
8.8 < X	0 (0)	14 (9)	17 (8)	19 (11)
**Ki-67 stage (dead of disease)**				
X ≤ 13%	82 (0)	201 (7)	108 (8)	36 (5)
13 < X	23 (5)	86 (27)	63 (20)	51 (17)

*RUL, right upper lobe; RML, right middle lobe; RLL, right lower lobe; LUL, left upper lobe; LLL, left lower lobe. lepidic predominant adenocarcinoma (LPA) is low-risk; papillary or acinar predomination adenocarcinoma (PPA or APA) is Intermediate-risk; SOL-MIP positive (SMP) is high-risk*.

### Survival Analysis

**Figure 1:**
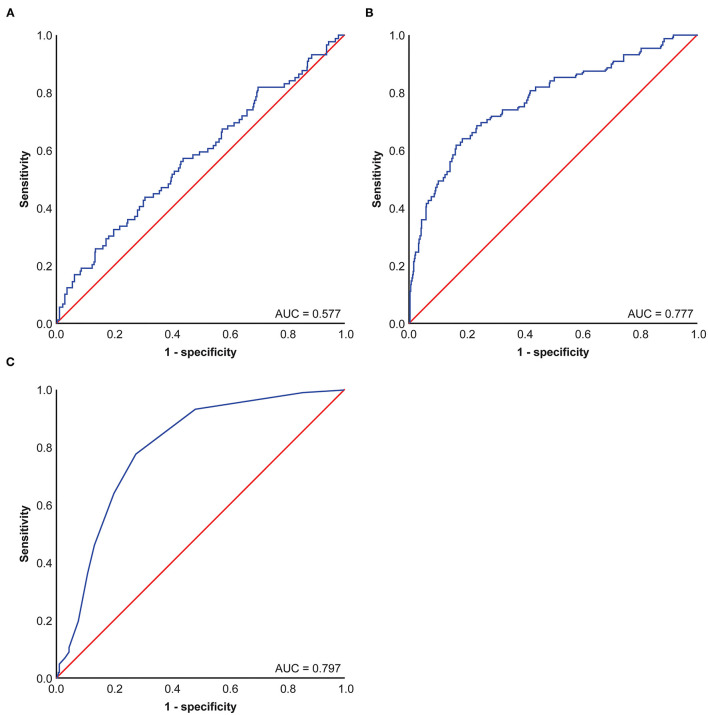
Receiver operating characteristic (ROC) Curve of carcinoembryonic antigen (CEA), KI-67, and age in patients of 650 patients with stage I pulmonary adenocarcinoma. **(A)** Age ROC Curve of 650 patients with stage I pulmonary adenocarcinoma, **(B)** CEA ROC Curve of 650 patients with stage I pulmonary adenocarcinoma, **(C)** KI-67 ROC Curve of 650 patients with stage I pulmonary adenocarcinoma.

**Figure 2:**
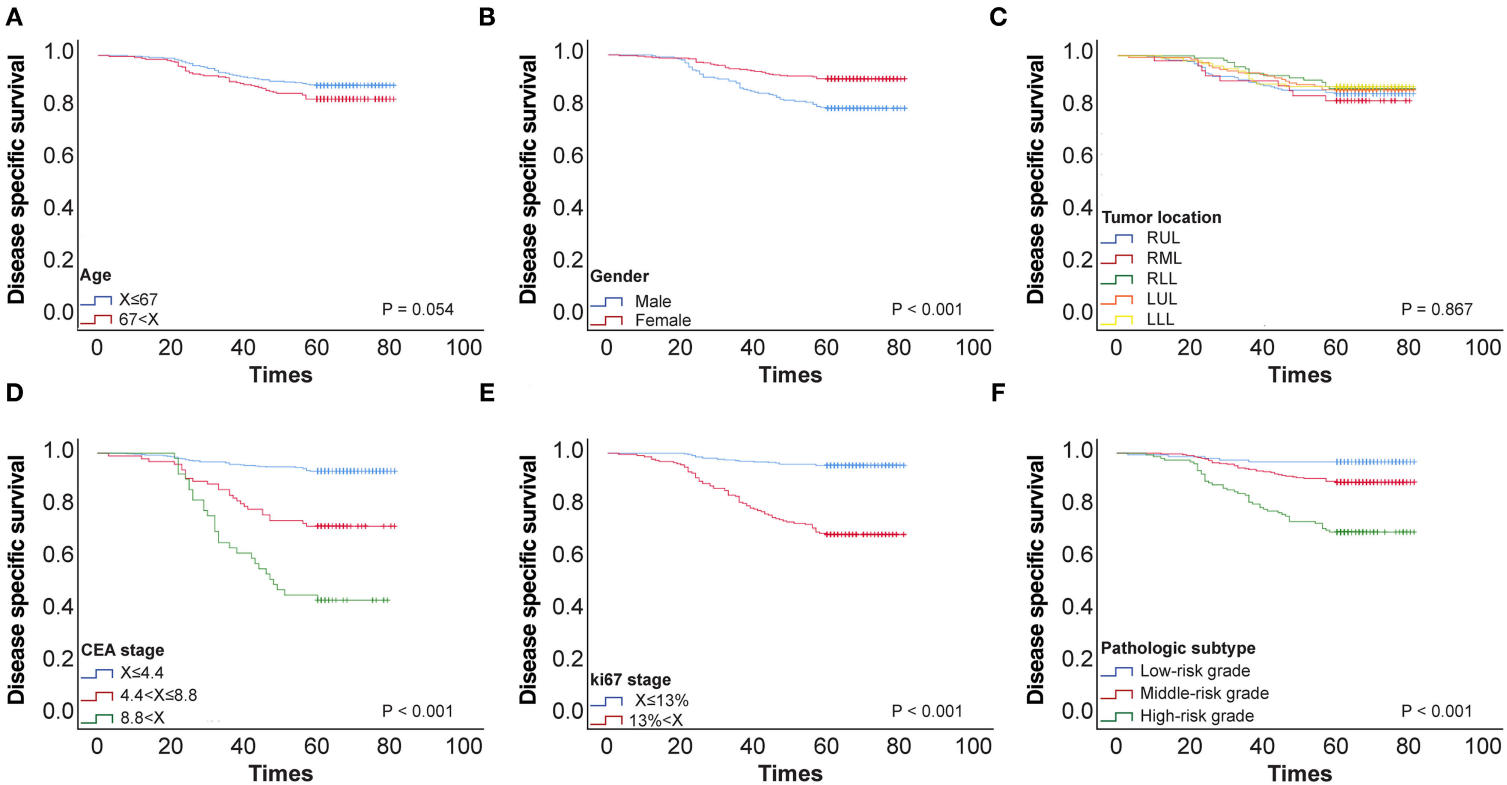
**(A)** 5-y disease-specific survival (DSS) curve of 650 patients with stage I pulmonary adenocarcinoma according to age. **(B)** 5-y DSS curve of 650 patients with stage I pulmonary adenocarcinoma according to Gender stage. **(C)** 5-y DSS curve of 650 patients with stage I pulmonary adenocarcinoma according to Tumor location. **(D)** 5-y DSS curve of 650 patients with stage I pulmonary adenocarcinoma according to CEA stage. **(E)** 5-y DSS curve of 650 patients with stage I pulmonary adenocarcinoma according to KI-67 stage. **(F)** 5-y DSS curve of 650 patients with stage I pulmonary adenocarcinoma according to Pathologic subtypes.

Using ROC curve analysis, potential cut-off values were obtained for CEA, patient age, and Ki-67 in 650 patients with stage I lung cancer. The AUC for age is 0.577, the AUC for CEA is 0.777, and the AUC for Ki-67 is 0.797. These cut-off points were 4.4 ng/ml for CEA, 67 years for age, and 13% for Ki-67 (Figure 1). Using Kaplan–Meier univariate analysis to analyze 650 patients with stage I lung cancer, neither age (*p*= 0.054) nor tumor location (*p* = 0.867) had prognostic significance. However, gender, pathologic subtype, CEA, and Ki-67 were predictors of 5-year DSS (Figure 2). Gender, pathologic subtype, CEA, and Ki-67 were thus introduced as categorical variables into Cox multivariate analysis (Table 2), and the female gender proved to be associated with better prognosis than male (HR: 0.591; 95% CI: 0.382–0.914; *p* = 0.018). There was no statistically significant difference in the risk associated with low-risk and intermediate-risk pathologic subtypes (*p* = 0.385). However, presence of high-risk tumor subtype was a significantly adverse prognostic factor compared with low-risk lesions (HR: 3.157; 95%CI: 1.198–8.324; *p* = 0.02). This means that the mere presence of a SOL or MIP component is a marker for adverse prognosis, despite the fact it is not the predominate pattern overall in the tumor. At the same time, according to Table 2, CEA and Ki-67 were found to be separate independent markers of poor prognosis for 5-y DSS (*p* < 0.001).

**Table 2:** Cyclooxygenase (COX) multivariable analysis of 5-year disease-specific survival (DSS) in 650 patients with stage I pulmonary adenocarcinoma.

**Variables**	**Multivariable**
	**HR (95% CI)**	***P*** **-value**
**Gender (male)**
Female	0.591 (0.382–0.914)	0.018
**Pathologic subtype (low-risk)**
Intermediate-risk	1.532 (0.585–4.008)	0.385
High-risk	3.157 (1.198–8.324)	0.020
**CEA stage (X** **≤4.4)**
4.4 < X ≤ 8.8	2.446 (1.443–4.146)	0.001
X > 8.8	6.060 (3.597–10.208)	<0.001
**Ki-67 stage (X≤13%)**
X > 13%	3.748 (2.195–6.401)	<0.001

*The risk grades characteristic of various tumor subtypes are as follows: lepidic predominant adenocarcinoma (LPA) is low-risk, papillary or acinar predomination adenocarcinoma (PPA or APA) is Intermediate-risk, and SOL-MIP positive (SMP) is high-risk*.

**Figure 3:**
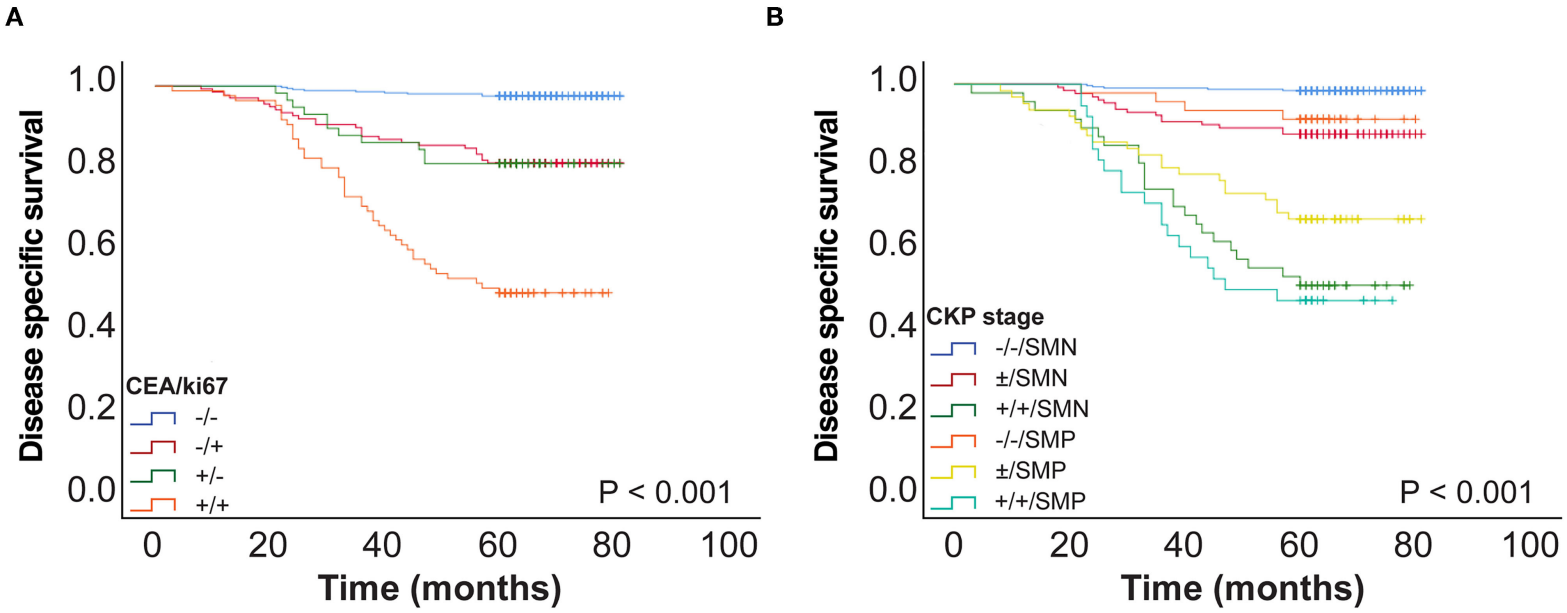
**(A)** CEA and Ki-67 combined as a novel factor designated CEA/Ki-67 and 5-year DSS curve of CEA/Ki-67 in 650 patients with stage I pulmonary adenocarcinoma. **(B)** 5-year DSS curve of CKP in 650 patients with stage I lung adenocarcinoma. SMN, SOL-MIP negative; SMP, SOL-MIP positive.

We then analyzed CEA and Ki-67 together as a combined risk factor (CEA/Ki-67). Cases where CEA ≤ 4.4 ng/ml and Ki-67 ≤ 13% were assigned a negative value designated as “–”, and CEA > 4.4 ng/ml and Ki-67 > 13% were assigned a positive value “+”. Kaplan–Meier univariate analysis (Figure 3) and Cox multivariate analysis were then performed (Table 3) in a total of 650 patients with stage I lung cancer. In comparison with CEA/Ki-67(–/–), the hazard ratios (HR) for CEA/Ki-67 (–/+), CEA/Ki-67 (+/–), and CEA/Ki-67 (+/+) showed a gradual rising trend, indicating these factors in combination more accurately predict prognosis than use of either alone.

**Table 3:** COX multivariable analysis of 5 y-DSS in 650 patients with stage I pulmonary adenocarcinoma.

**Variables**	**Multivariable**
	**HR (95% CI)**	***P*** **-value**
**Gender (male)**		
Female	0.706 (0.460–1.085)	0.112
**Pathologic subtype (low-risk)**		
Intermediate-risk	1.663 (0.643–4.302)	0.295
High-risk	3.157 (1.178–8.039)	0.022
**CEA/Ki-67 stage (–/–)**		
–/+	6.218 (2.845–13.592)	*P* <0.001
+/–	6.781 (2.772–16.590)	*P* <0.001
+/+	18.298 (8.597–38.948)	*P* <0.001

*Carcinoembryonic antigen (CEA) ≤ 4.4 ng/ml and Ki-67 ≤ 13% were assigned a negative value “–”, and CEA > 4.4 ng/ml and Ki-67 > 13% were assigned a positive value “+”. CI, confidence interval; HR, hazard ratio*.

In view of the CEA/Ki-67 HR in Table 3, CEA/Ki-67 values “–/+” and “+/–” were grouped together and designated “±”. Similarly, according to the HR and *p*-value of the pathologic subtype (as in Table 3), we combined low-risk and intermediate-risk pathologic subtypes and designated them as SOL-MIP negative. The high-risk group retained the designation SOL-MIP positive. We next combined values for CEA, Ki-67, and pathologic subtype which yielded a novel prognostic value designated in this study as CKP. CKP includes six categories: –/–/ SOL-MIP negative, ±/ SOL-MIP negative, +/+/ SOL-MIP negative, –/–/ SOL-MIP positive, ±/ SOL-MIP positive, and +/+/ SOL-MIP positive. According to our findings, CEA > 4.4 ng/ml, Ki-67 > 13%, and SOL-MIP positive were found to confer independent adverse prognosis. Particularly, “–/–/ SOL-MIP negative” means that there are no risk factors; “±/ SOL-MIP negative, –/–/ SOL-MIP positive” indicates there is one factor for risk; “±/SOL-MIP positive, +/+/SOL-MIP negative” means there are two risk factors; and, similarly, “+/+/SOL-MIP positive” means that there are three risk factors. According to the result of Kaplan–Meier univariate analysis (Figure 3B), “–/–/SOL-MIP negative, ±/SOL-MIP negative, and –/–/SOL-MIP positive” were then assigned to one group, and this group was defined as the low-risk group. “±/SOL-MIP negative, +/+/SOL-MIP negative, and +/+/SOL-MIP positive” were assigned to another group defined as high-risk. In summary, this means that if there is one risk factor or less for a given tumor, it is defined as low-risk; if there are two or more risk factors, the tumor is defined as high-risk.

**Figure 4:**
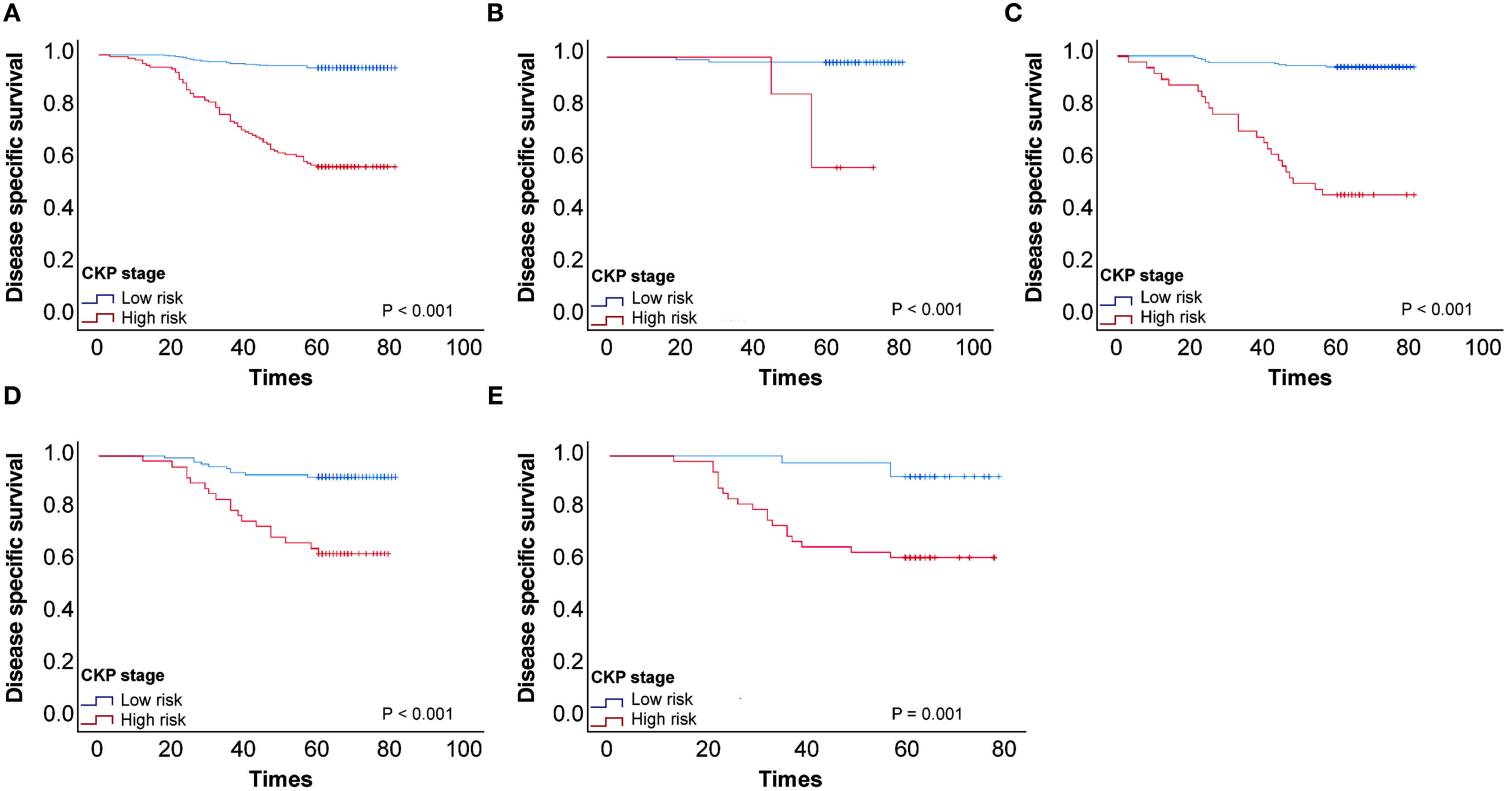
**(A)** Kaplan–Meier univariate analysis with CEA-Ki-67-pathologic subtype (CKP) values for patients with stage I pulmonary adenocarcinoma, **(B)** Kaplan–Meier univariate analysis with CKP values for stage I sub-stages IA1, **(C)** Kaplan–Meier univariate analysis with CKP values for stage I sub-stages IA2, **(D)** Kaplan–Meier univariate analysis with CKP values for stage I sub-stages IA3, **(E)** Kaplan–Meier univariate analysis with CKP values for stage I sub-stages IB.

**Figure 5:**
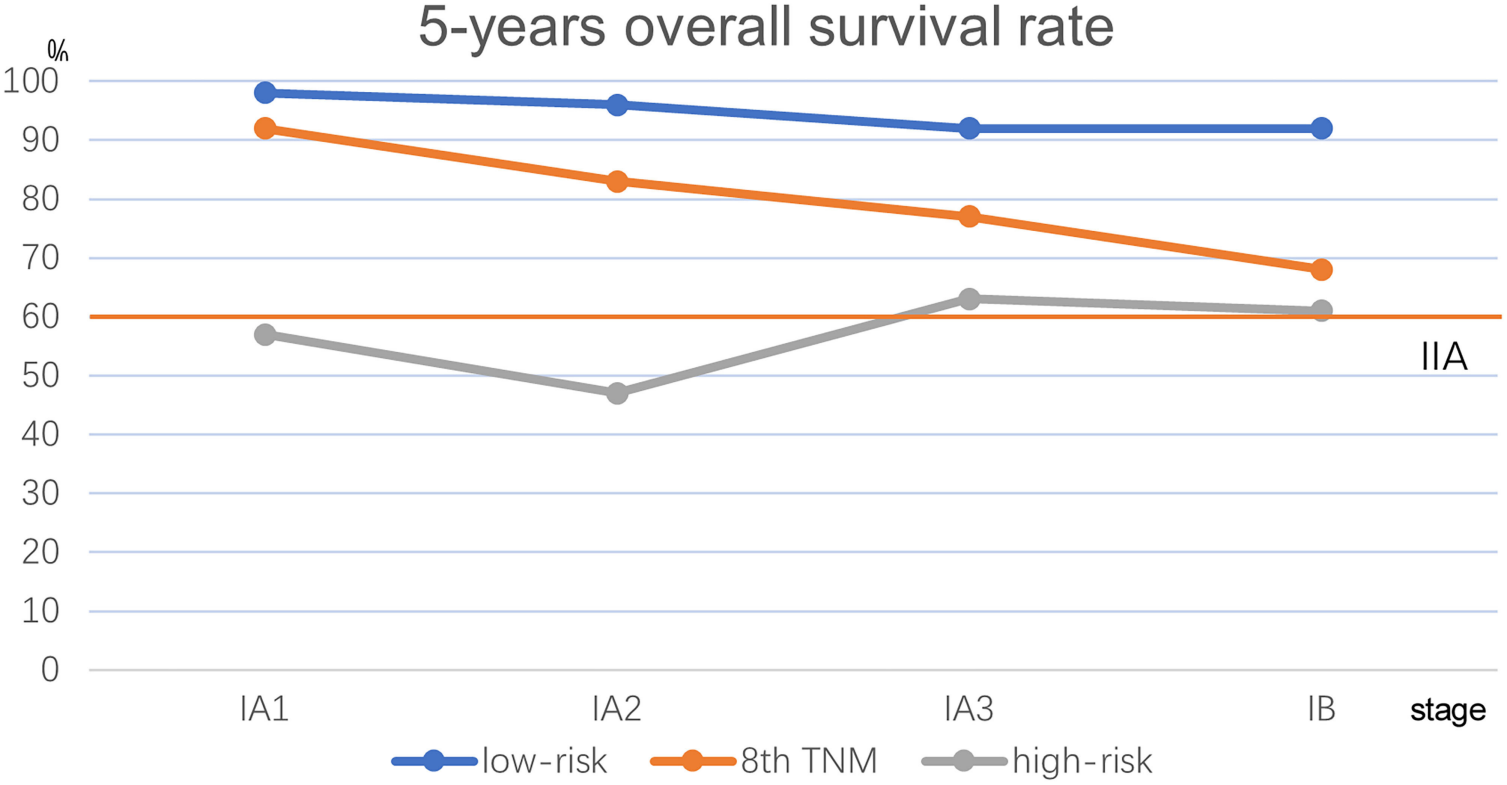
Line chart comparison the 8th TNM classification and TNM-CKP value. Red line depicts the 5-year overall survival rate for stage IIA.

We performed Kaplan–Meier univariate analysis with CKP values for pulmonary adenocarcinoma stage I, sub-stages IA1, IA2, IA3, and IB (Figure 4). This demonstrated that CKP value is the factor which most significantly reflects stage I sub-stage risk (*p* < 0.001). Five-year survival rates were then compared based on the eighth edition TNM classification and the TNM-CKP value (Table 4), and a corresponding line chart was drawn (Figure 5). We found that regardless of which stage I sub-stages were compared, if a given substage was low-risk for CKP value, the prognosis was similar to stage I A1. However, if a given substage had a high-risk CKP value, the prognosis was similar to stage II combining the 8th TNM classification and CKP value yields a more precise evaluation of the prognosis.

**Table 4:** Five-year survival rate comparing the eighth edition stage and CEA-Ki-67-pathologic subtype (CKP) value in patients with stage I pulmonary adenocarcinoma (*n* = 650).

**CKP value (survival rate)**	**IA1 (***n*** = 105)**	**IA2 (***n*** = 287)**	**IA3 (***n*** = 171)**	**IB (***n*** = 87)**
Low-risk group	98 (98%)	242 (96%)	123 (92%)	38 (92%)
High-risk group	7 (57%)	45 (47%)	48 (63%)	49 (61%)
Eighth edition survival	92%	83%	77%	68%

*Low-risk group, “–/–/SMN; ±/SMN and –/–/SMP”; high-risk group, “±/SMN, +/+/SMN and +/+/SMP”; CKP value, CEA-Ki-67-pathologic subtype value. SMN, SOL-MIP negative; SMP, SOL-MIP positive*.

## Discussion

To the best of our knowledge, this study is the first to use CEA, Ki-67, and pathologic subtype in concert, yielding a novel combined prognostic factor designated CEA-Ki-67 - Pathologic subtype value (CKP value) in patients with stage I pulmonary adenocarcinoma. Adjuvant therapy after surgery for stage I pulmonary adenocarcinoma is controversial and is not currently uniformly recommended. In the present study, patients with stage IA1, IA2, IA3, or IB tumors and a low-risk CKP value had a prognosis which was similar to stage IA1. This indicates that these tumors are prognostically similar to stage I tumors and that adjuvant therapy after surgery is not warranted. Conversely, patients with a high-risk CKP value have a prognosis similar to stage II, and adjuvant therapy may therefore be indicated. As such, the CKP value may be utilized as an adjunct to usual TNM staging for evaluation of stage I pulmonary adenocarcinoma to best determine which patients should be treated as stage I and which patients' tumors warrant treatment as stage II.

Previous studies have demonstrated that the Ki-67 proliferation index is an accurate gage of prognosis in NSCLC, and that Ki-67 > 25% is associated with poor prognosis in stage I-IV pulmonary adenocarcinoma (14). In the current study, we found Ki-67 > 13% is an independent adverse prognostic marker in stage I pulmonary adenocarcinoma. However, at this time, there is no widely accepted cut-off point which definitively specifies prognosis in patients with NSCLC. Many previous single-center studies have in fact used different Ki-67 cut-off points (15). Several factors contribute to this lack of specificity for Ki-67 prognostic grading. First, a majority of the studies which have evaluated Ki-67 either did not have sufficient data or were a single-center design. Second, Ki-67 is evaluated with immunohistochemistry which yields a percentage of tumor cell staining, and this is, in some measure, unavoidably subjective. Third, some studies have shown that Ki-67 values are associated with prognosis which varies with different histologic subtypes, differentiation status, and cancer stage (16). However, in this study, statistical analysis was performed in stage I lung adenocarcinoma (stage as assigned by traditional TNM), which excludes the influence of lymph node status, distant metastasis, or tumor type. Evaluation of Ki-67 was performed by two pathologists to minimize the influence of subjectivity. Ultimately, we found that in this cohort, Ki-67 > 13% was an independent adverse prognostic factor.

Arrieta et al. observed that a high serum level of CEA is a marker for development of brain metastasis and is generally associated with poor prognosis, and surface expression of CEA on tumor cells may be a part of the pathophysiologic mechanism of invasion of the central nervous system (17). In this study, we also found that CEA is an independent adverse prognostic factor in patients with stage I pulmonary adenocarcinoma (4.4 < CEA ≤ 8.8: HR: 2.446; 95%CI: 1.443–4.146; *p* = 0.001; CEA > 8.8: HR: 6.060; 95% CI: 3.597–10.208; *p* < 0.001). We also observed a relationship wherein increasingly high CEA values were associated with increasingly poor prognosis. In addition, we found that combining CEA and Ki-67 values together in patients with stage I pulmonary adenocarcinoma yields a more accurate prediction of prognosis. Patients with simultaneous values of CEA > 4.4 ng/ml and Ki-67 > 13% had a significantly poorer 5-year DSS than those with normal values (HR: 18.298; 95% CI: 8.597–38.948; *p* < 0.001).

Many studies divide pathologic subtypes of pulmonary carcinoma into low-risk (LPA), intermediate-risk (PPA or APA), and high-risk (SPA or MPA). However, in this study, we define SOL-MIP positive as high-risk. Many studies have shown that an SOL or MIP histologic component is significantly associated with poor relapse-free survival and prognosis (18, 19). Relevant literature also indicates that patients with MIP or SOL components are not unlikely to have microvascular invasion or lymph node micrometastasis (20, 21). Therefore, if only SPA or MPA cases are considered as a high-risk pathologic subtype, categorization of SOL-MIP positive into low or intermediate-risk group may engender bias. However, in our study, we categorize SOL-MIP positive as high-risk. This may serve to reduce bias and explain why there was no significant statistical difference in risk in the intermediate-risk and low-risk groups (*p* = 0.385), while at the same time, there was a significant statistical difference in risk between high-risk and low-risk grades (*p* = 0.02; HR = 3.157; 95% CI: 1.198–8.324) in the multivariate analysis of prognosis in this study.

The eighth edition of the TNM classification, published in 2017, analyzed tumor size, lymph node status, and distant metastasis in 94,708 patients with lung cancer who were diagnosed between 1999 and 2010. This number of evaluated patients is very large (2). However, the prognostic factors it takes into consideration are few, especially in patients with stage I pulmonary adenocarcinoma. In stage I, there are, by definition, no lymph node metastasis and no distant metastasis, and, as such, the only prognostic factor is tumor size. Tumor size alone has limitations for predicting patient prognosis. Our current study suggests that TNM staging might best be improved by switching from simple staging based on anatomic features alone to comprehensive staging with the use of adjunct biomarkers and/or interpolation of histologic subtype.

This study has several limitations. It is a single-center retrospective study, and the sample size is relatively small. In addition, due to the limitations of data collection, we did not get enough disease-free survival (DFS). These findings will require validation in multicenter studies to further evaluate application of CKP value in patients with stage I lung adenocarcinoma. This will support either the need for further treatment due to higher risk, or conversely assign patients with stage I adenocarcinoma to a prognostic category which represents likelihood of low-grade stage I behavior. The use of the CKP value as an adjunct to TNM staging may thus refine assessment of stage I patient outcomes.

## Conclusion

Carcinoembryonic antigen >4.4 ng/ml, KI-67 > 13%, and solid-micropapillary positivity are independent risk factors for 5-year DSS in stage I pulmonary adenocarcinoma. The use of this novel CKP value as an adjunct to TNM staging allows more comprehensive staging of patients with stage I pulmonary adenocarcinoma and identifies those who are at higher risk. This allows better evaluation of patients who may require adjuvant chemotherapy to improve survival time, and those unlikely to benefit from adjuvant therapy.

## Data Availability Statement

The original contributions presented in the study are included in the article/supplementary material, further inquiries can be directed to the corresponding author/s.

## Ethics Statement

The Institutional Review Board of The First Affiliated Hospital of China Medical University approved this retrospective study (2021-99). Written informed consent from individual patients was waived because the study was a retrospective review.

## Author Contributions

SX and DY designed the research. DY and YS reviewed the medical records and collected and analyzed the data. DY and MM wrote the manuscript. All authors contributed to the article and approved the submitted version.

## Funding

This work was supported by the Scientific Research of Department of Science and Technology of Liaoning Province (grant number 2017225035).

## Conflict of Interest

The authors declare that the research was conducted in the absence of any commercial or financial relationships that could be construed as a potential conflict of interest.

## Publisher's Note

All claims expressed in this article are solely those of the authors and do not necessarily represent those of their affiliated organizations, or those of the publisher, the editors and the reviewers. Any product that may be evaluated in this article, or claim that may be made by its manufacturer, is not guaranteed or endorsed by the publisher.

## Abbreviations

DSS: disease specific survival
NSCLC: non-small cell lung cancer
CEA: carcinoembryonic antigen
LPA: lepidic predominant invasive adenocarcinoma
PPA: papillary predominant invasive adenocarcinoma
APA: acinar predominant invasive adenocarcinoma
MIP: micropapillary
AIS: adenocarcinoma *in situ*
MIA: minimally invasive adenocarcinoma
CKP value: CEA-Ki-67-pathologic subtype value.
